# In the letting go

**DOI:** 10.1017/S1478951524002116

**Published:** 2025-01-21

**Authors:** Antonio Yaghy

**Affiliations:** Department of Ophthalmology and Visual Sciences, University of Massachusetts Chan Medical School, Worcester, MA, USA

The morning light crept across Maria’s hospital bed – tentative, hesitant, much like Isabella’s own thoughts as she watched her mother sleep. Tubes and wires created a complex web around her once-dancing spirit, while monitors counted heartbeats like lonely metronomes in this clinical silence. Through the window, autumn leaves danced on the breeze, free and wild, so unlike her mother’s now-confined existence.

“Stage four choroidal melanoma … spread extensively ….” The oncologist’s words from that morning hung in the air, sharp and heavy. Isabella closed her eyes, remembering her mother teaching her to knead bread in their small bakery, strong hands guiding her own through the familiar motions. How could those same hands now lie so still?

“No.” Marco’s voice cut through the silence as he burst into the room, startling Isabella from her reverie. “We need to try another round of chemo. There must be other trials, experimental treatments—”

“Marco—” Isabella began, but he was already pacing, his suit jacket thrown carelessly over a chair, tie loosened like he was trying to breathe through a choking grip.

“I’ve been researching. There’s a facility in Houston, they’re doing revolutionary work with—”

“Enough.” The word, barely a whisper, came from their mother. Maria’s eyes fluttered open, finding her children with the same warmth that had guided them through skinned knees and broken hearts. “Sit with me, mis amores.”

They gathered close, Marco’s resistance melting as their mother’s fragile hand reached for his. Her face held the afternoon light like a precious secret, even as illness had written its harsh poetry across her features.

“I remember,” Maria began, her voice thin but clear, “when you were small, Marco, you tried to catch the wind in a jar.” A slight smile touched her lips. “You were so determined, running through the yard with your mason jar held high.”

Marco’s shoulders shook. “You told me some things aren’t meant to be captured,” he whispered. “That their beauty lies in their freedom.”

Isabella watched her brother’s face crumple, the defensive armor of denial finally cracking. Maria squeezed his hand, her next words careful and measured: “The time for running with jars has passed, mi hijo.”

The palliative care discussion came gradually, like evening settling into night. Dr. Chen, the palliative care specialist, didn’t push or prescribe – she listened. She sat with them through tears and questions, through Marco’s angry outbursts and Isabella’s quiet fears.

“What does giving up look like to you?” Dr. Chen asked one afternoon, after Marco had listed every experimental treatment from Boston to Beijing.

The question caught him off-guard. He stood by the window, Philadelphia’s skyline a glittering backdrop to their pain. “Like failure,” he admitted finally. “Like I’m not trying hard enough to save her.”

“And what does love look like?” Dr. Chen’s gentle query hung in the air.

It was Maria who answered, her voice stronger than it had been in days: “Love looks like letting your children see you as human. Like showing them that even in ending, there is grace.”

The transition wasn’t a surrender but a slow waltz – two steps forward, one step back. They moved their mother to the palliative care unit on a Thursday, the day’s weak winter sunlight somehow softer here than in the oncology ward. The walls welcomed family photos, and the strict visiting hours dissolved into a gentle flow of presence and care.

Isabella brought her mother’s recipe box from home, and they spent hours recording the secret ingredients of family favorites. “A pinch of cumin,” Maria would insist, while Isabella frantically scribbled measurements that had only ever existed in her mother’s muscle memory.

“But how will I know when it’s right?” Isabella asked one day, frustrated by the imprecision.

Maria touched her daughter’s cheek. “You’ll feel it here,” she said, then moved her hand to her heart. “And here. Just like you’ll know how to live when I’m gone.”

Marco began spending his lunch breaks at the unit, his corporate laptop incongruous against the homey surroundings. He would sit beside their mother, typingone-handed, the other hand always holding hers. Sometimes they talked; often they didn’t. The silence between them grew comfortable, like a well-worn blanket.

The hard days came too – hours filled with pain that even the best medications couldn’t completely mask. During one particularly difficult night, as Maria’s breath rattled and caught, Marco broke.

“I can’t—” he choked out, backing toward the door. “I can’t watch—”

Isabella caught him in the hallway, her own tears mixing with his as they clung to each other. “We can,” she whispered fiercely. “We must. Because she did it for us, every skinned knee, every broken heart. She stayed.”

Time became fluid, measured not in hours but in moments: the afternoon Maria taught her grandchildren to fold paper cranes from her bed, her movements slow but precise; the morning the entire palliative care team gathered to celebrate her seventieth birthday, the room filled with more joy than sorrow; the quiet evening when she told them stories of their father, gone too young but never forgotten.

“Life,” Maria told them during one of her clearer moments, “is like baking bread. You combine what you have, you work with love, you wait through the rising and falling. And in the end, what matters is not how long it lasted, but how much love you kneaded into the dough.”

The final days arrived like twilight – gradually, then all at once. Maria’s words became fewer, but her eyes spoke volumes. They took turns reading to her, playing her favorite boleros, holding her hand as each breath became a prayer, separated by expanding moments of sacred silence.

On her last morning, as dawn painted the sky in watercolor strokes of pink and gold, Maria opened her eyes with unexpected clarity. “My loves,” she whispered, “thank you for letting me teach you this final lesson – that love means knowing when to hold tight, and when to open your hands to the wind.”

She slipped away as she had lived – surrounded by love, her children’s hands in hers, the scent of cinnamon and warmth from the coffee Isabella had brought lingering in the air. In the profound silence that followed, Isabella and Marco held each other, their grief and gratitude intertwining like the complex braids of the challah bread their mother had once taught them to make.

Later, they would find among their mother’s things a note, written in her familiar slanting script: “Life is not measured in years but in loves. My cup has overflowed. Let me show you how to die the way I lived – with grace, with dignity, and with an open heart.”

In choosing palliative care, they had not given up on living – they had chosen to honor life’s final chapter with the same love and attention their mother had given to all the chapters before. In the end, they learned that sometimes the greatest courage lies not in fighting against the inevitable, but in embracing it with grace, allowing love to light the way through the darkness of letting go.

